# Pathway-Level Convergence Between Dynamic Plasma miRNAs and Endometrial Biological Processes During the Human Peri-Implantation Window

**DOI:** 10.3390/ijms27052414

**Published:** 2026-03-05

**Authors:** Chun-I Lee, An Hsu, Yu-Jen Lee, En-Hui Cheng, Chi-Ying Lee, Pin-Yao Lin, Maw-Sheng Lee, Chung-I Chen, Tzu-Ning Yu, Tiffany Wang, Cai-Yun Wang, Shi-Ting Lin, Jung-Hsuan Yang, Hui-Ling Hsu, Eric Pok Yang, Tsung-Hsien Lee

**Affiliations:** 1 Division of Infertility, Lee Women’s Hospital, Taichung 406, Taiwan; adoctor0402@gmail.com (C.-I.L.); jellylin0607@gmail.com (P.-Y.L.); msleephd@gmail.com (M.-S.L.); cci1959@yahoo.com.tw (C.-I.C.); ningsyu@gmail.com (T.-N.Y.); 2 Department of Obstetrics and Gynecology, School of Medicine, Chung Shan Medical University, Taichung 402, Taiwan; 3 Department of Obstetrics and Gynecology, Chung Shan Medical University Hospital, Taichung 402, Taiwan; 4 Inti Labs, Hsinchu 302, Taiwan; bernett@intilabs.com (A.H.); tiffany@intilabs.com (T.W.); irenew989@gmail.com (C.-Y.W.); phyllislin@intilabs.com (S.-T.L.); sandyyang@intilabs.com (J.-H.Y.); scss00991@gmail.com (H.-L.H.); 5 Genetic Diagnosis Laboratory, Lee Women’s Hospital, Taichung 406, Taiwan; yujen1027@gmail.com (Y.-J.L.); enhuicheng@gmail.com (E.-H.C.); saminmon@gmail.com (C.-Y.L.); 6 Department of Post-Baccalaureate Medicine, College of Medicine, National Chung Hsing University, Taichung 403, Taiwan; 7 Institute of Bioinformatics and Structural Biology, National Tsing Hua University, Hsinchu 300, Taiwan; 8 Institute of Medicine, Chung Shan Medical University, Taichung 402, Taiwan

**Keywords:** peri-implantation window, microRNA, endometrium, plasma miRNA, pathway enrichment, temporal dynamics, implantation biology, systems biology

## Abstract

The peri-implantation window is a tightly regulated temporal phase during which the human endometrium undergoes coordinated molecular remodeling to establish receptivity. MicroRNAs (miRNAs) contribute to implantation-related processes; however, whether dynamic endometrial regulatory signals are functionally reflected in circulation within a defined temporal framework remains unclear. We hypothesized that although individual miRNA identities differ between endometrial tissue and plasma, temporally regulated miRNAs in both compartments may exhibit overlap at the level of enriched biological pathways during the peri-implantation window. To test this hypothesis, we performed time-resolved small RNA sequencing on paired endometrial and plasma samples collected from 62 participants across progesterone exposure days P+3 to P+7 in hormonally controlled cycles. Temporal modeling identified 27 dynamic miRNAs in endometrial tissue and 17 in plasma (FDR < 0.05). Despite limited overlap at the individual miRNA level, functional enrichment analysis revealed recurrent overlap in apoptosis-, cell cycle-, aging-, inflammatory-, and metabolic-related pathways across compartments. Four miRNAs exhibited concordant directional temporal trends between tissue and plasma with moderate correlation coefficients. These findings suggest that dynamic miRNA-associated enrichment patterns during the peri-implantation window may exhibit pathway-level overlap despite divergence in specific molecular identities. This temporally aligned integrative framework provides a pathway-centric perspective for interpreting cross-compartment miRNA-associated temporal patterns and supports a hypothesis-generating systems-level view of human implantation biology.

## 1. Introduction

The peri-implantation window represents a tightly regulated temporal phase during early pregnancy in which the endometrium transitions into a receptive state capable of supporting embryo attachment and invasion. This interval, commonly referred to as the window of implantation (WOI), is generally considered to occur between three and seven days after progesterone administration (P+3 to P+7) in hormone replacement therapy (HRT) cycles [1,2]. Successful implantation requires coordinated endocrine signaling, epithelial–stromal remodeling, immune modulation, and metabolic reprogramming within a precisely defined timeframe [3,4,5]. Increasing evidence suggests that implantation is not governed by isolated molecular events but rather by temporally orchestrated regulatory networks operating across multiple biological pathways.

MicroRNAs (miRNAs) are small non-coding RNAs of approximately 20–30 nucleotides that post-transcriptionally regulate gene expression and contribute to fine-tuning cellular processes. In the human endometrium, miRNAs have been implicated in proliferation, differentiation, decidualization, epithelial plasticity, and receptivity-related remodeling [6,7,8,9,10,11]. Importantly, miRNA expression patterns vary dynamically across the menstrual cycle, reflecting temporal modulation of endometrial physiology [6,8,9]. Recent studies emphasize that the biological significance of miRNA activity during implantation may be more meaningfully interpreted at the level of enriched functional pathways rather than individual miRNA species alone [8,9,10,11]. Such pathway-centric perspectives are particularly relevant in dynamic biological systems where coordinated regulation may occur despite molecular heterogeneity.

In parallel with tissue-based investigations, circulating cell-free miRNAs in peripheral blood have attracted growing interest due to their relative stability and accessibility [12,13,14]. Temporal changes in plasma miRNA expression during the peri-implantation stage and early pregnancy have been reported [12,13], suggesting potential systemic reflections of reproductive remodeling. However, most prior studies have examined endometrial tissue or circulating miRNAs independently. Whether dynamic regulatory signals observed in the endometrium are functionally mirrored in circulation within a defined temporal framework remains insufficiently characterized.

Notably, cross-compartment comparisons have largely focused on individual overlapping miRNA species. Yet, dynamic biological remodeling may manifest as convergence at the level of functional pathways even when specific molecular identities differ between compartments. A systematic evaluation of temporally aligned endometrial and plasma miRNA profiles within the same cohort has not been comprehensively performed. Clarifying whether pathway-level enrichment patterns exhibit cross-compartment convergence during the peri-implantation window would provide important insight into systemic coordination of implantation-related processes.

We therefore hypothesized that although individual miRNA identities may differ between endometrial tissue and plasma, temporally regulated miRNAs in both compartments would exhibit overlap at the level of enriched biological pathways during the peri-implantation window. To test this hypothesis, we performed time-resolved small RNA sequencing on paired endometrial and plasma samples collected from P+3 to P+7 in hormonally controlled cycles. Through integrative temporal modeling and pathway enrichment analysis, we examined cross-compartment relationships of dynamic miRNA-associated functional categories. This study establishes a temporally aligned framework for evaluating pathway-level convergence of miRNA regulation during human peri-implantation remodeling.

## 2. Results

### 2.1. Study Design and Cohort Characteristics

To investigate whether circulating miRNAs reflect temporal regulatory changes occurring in the endometrium during the peri-implantation window, we established a temporally aligned sampling framework within hormonally controlled cycles. A total of 62 participants undergoing HRT cycles were stratified into five groups according to progesterone exposure day (P+3 to P+7), enabling reconstruction of a defined peri-implantation time window.

At each time point, paired endometrial tissue and plasma samples were collected. Baseline clinical characteristics, including age, body mass index (BMI), FSH, LH, estrogen, progesterone levels, and endometrial thickness, showed comparable distributions across groups (Table 1; Supplementary Figures S1 and S2). This HRT-based controlled design minimizes inter-cycle variability and enables direct comparison of temporally aligned miRNA expression patterns between endometrial tissue and plasma.

### 2.2. miRNA Abundance and Distribution in Endometrial Tissue and Plasma

Small RNA sequencing was performed on all 62 paired samples to profile miRNA expression during the peri-implantation window, followed by miRBase annotation. Sequencing results are summarized in Supplementary Table S1. After adaptor trimming and quality filtering, the average number of human genome-mappable reads was 4,037,654 for endometrial tissue samples and 6,230,647 for plasma samples. In endometrial tissue, an average of 1,711,055 reads (43.1% of mappable reads) were annotated as miRNAs, corresponding to 336 distinct miRNAs. In contrast, plasma samples yielded an average of 590,628 miRNA reads (9.5% of mappable reads) and 205 distinct miRNAs.

Overall, endometrial tissue showed a higher proportion of miRNA reads and a greater number of detectable miRNA species than plasma samples. These data describe differences in miRNA abundance and diversity between compartments and provide a quantitative basis for subsequent comparative analyses.

### 2.3. Temporal miRNA Expression Patterns in Endometrial Tissue During the Peri-Implantation Window

**Figure 1:**
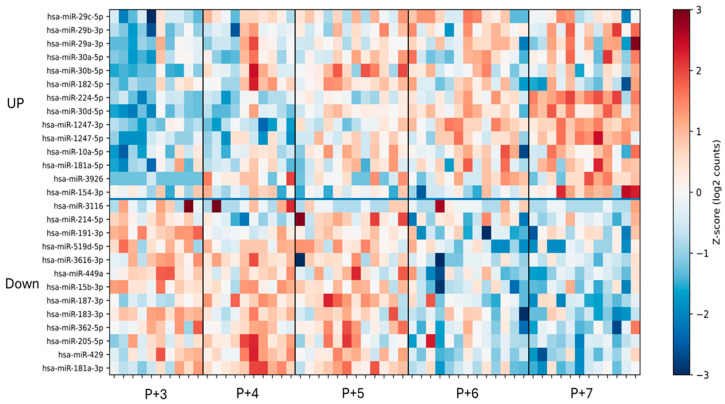
Heatmap of temporally dynamic miRNAs in endometrial tissue from P+3 to P+7. The heatmap displays miRNAs exhibiting significant temporal changes identified by DESeq2 analysis (adjusted *p* < 0.05) across five consecutive time points (P+3 to P+7). Hierarchical clustering was performed based on similarities in expression patterns. The upper cluster represents miRNAs with progressively increased expression over time, whereas the lower cluster includes miRNAs showing a decreasing trend. Expression values were log2-transformed and standardized using Z-score normalization. Red indicates relatively higher expression levels, and blue indicates lower expression levels.

We examined temporally dynamic miRNA expression patterns in endometrial tissue across the peri-implantation window (P+3 to P+7). Small RNA sequencing identified 27 temporally dynamic miRNAs in endometrial tissue samples (adjusted *p* < 0.05). Hierarchical clustering and heatmap visualization demonstrated distinct temporal expression patterns among these miRNAs (Figure 1). Based on their trajectories from P+3 to P+7, the temporally dynamic miRNAs were grouped into two major patterns: progressively increasing and progressively decreasing expression across the time course.

Correlation analysis between temporally dynamic endometrial miRNAs and clinical parameters revealed significant associations with luteinizing hormone (LH) levels, estrogen levels, and endometrial thickness (Supplementary Figure S3; Supplementary Table S2). Comparison of miRNA profiles between participants with and without a history of pregnancy did not show significant differences (Supplementary Table S3).

### 2.4. Functional Enrichment of Temporally Dynamic miRNAs in Endometrial Tissue

**Figure 2:**
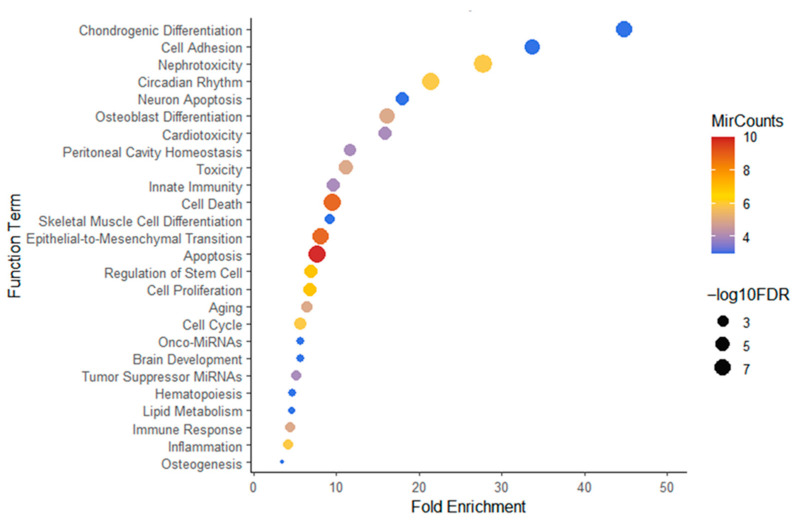
Functional enrichment of temporally dynamic endometrial miRNAs identified from P+3 to P+7. Functional enrichment analysis was performed using TAM 2.0 on miRNAs exhibiting significant temporal dynamics (DESeq2, adjusted *p* < 0.05). The x-axis represents fold enrichment relative to background expectation. Bubble color indicates the number of miRNAs associated with each functional category (red denotes a higher number of miRNAs, whereas blue denotes fewer miRNAs). Bubble size corresponds to statistical significance (–log10 FDR), with larger bubbles indicating greater statistical significance.

To explore biological themes associated with temporally dynamic miRNA expression in endometrial tissue, predicted target genes were analyzed using TAM 2.0 for functional enrichment. Dynamic miRNAs were enriched in multiple biological categories (FDR < 0.05), including apoptosis, cell death, epithelial-to-mesenchymal transition (EMT), cell cycle regulation, inflammation, lipid metabolism, and differentiation-related pathways (Figure 2; Supplementary Table S4).

Among the enriched categories, Chondrogenic Differentiation and Cell Adhesion showed higher fold enrichment values. In contrast, Apoptosis, Cell Death, and EMT included the largest numbers of contributing miRNAs. These enrichment results indicate that temporally dynamic miRNAs in endometrial tissue are associated with pathways related to cellular survival and structural remodeling during the peri-implantation window.

### 2.5. Dynamic Plasma miRNAs and Associated Functional Categories During the Peri-Implantation Window

**Figure 3:**
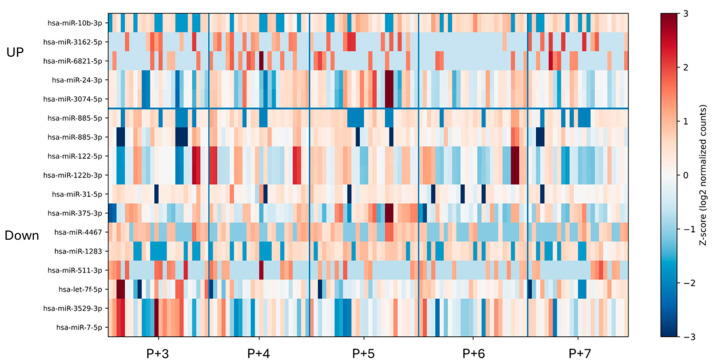
Heatmap of temporally dynamic plasma miRNAs across progesterone exposure days (P+3 to P+7). The heatmap displays plasma miRNAs exhibiting significant temporal changes identified by DESeq2 analysis (adjusted *p* < 0.05) across five consecutive progesterone exposure days (P+3 to P+7). Hierarchical clustering was performed based on expression pattern similarity. A total of 17 temporally dynamic miRNAs were identified. The upper cluster represents miRNAs with progressively increased expression over time, whereas the lower cluster includes miRNAs showing decreasing trends. Expression values were log2-transformed and standardized using Z-score normalization. Red indicates relatively higher expression levels and blue indicates lower expression levels.

We next examined whether circulating plasma miRNAs exhibited temporal dynamics across the peri-implantation window. Small RNA sequencing identified 17 temporally dynamic miRNAs in plasma samples collected from P+3 to P+7 (Figure 3). Compared with endometrial tissue, plasma miRNAs displayed distinct temporal expression profiles and limited overlap at the individual miRNA level.

**Figure 4:**
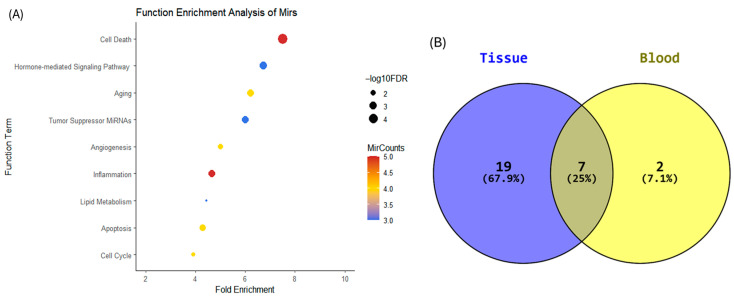
Functional enrichment of temporally dynamic plasma miRNAs and overlap with endometrial tissue. (**A**) Functional enrichment analysis of temporally dynamic plasma miRNAs identified across progesterone exposure days (P+3 to P+7) using TAM 2.0 (DESeq2; adjusted *p* < 0.05). The x-axis represents fold enrichment relative to background expectation. Bubble color indicates the number of miRNAs associated with each functional category (red denotes a higher number of miRNAs, whereas blue denotes fewer miRNAs). Bubble size corresponds to statistical significance (−log10 FDR), with larger bubbles indicating greater statistical significance. (**B**) Venn diagram illustrating the overlap of enriched functional categories between plasma and endometrial tissue miRNAs. Shared functional terms between the two compartments are indicated.

Functional enrichment analysis indicated that these temporally dynamic plasma miRNAs were enriched (FDR < 0.05) in multiple functional categories, including Hormone-Mediated Signaling Pathway, Lipid Metabolism, Apoptosis, Cell Cycle, Cell Death, Inflammation, Aging, Tumor Suppressor miRNAs, and Angiogenesis (Figure 4A). Comparison of enriched functional categories between plasma and endometrial tissue revealed seven shared terms: Cell Death, Apoptosis, Cell Cycle, Aging, Inflammation, Tumor Suppressor miRNAs, and Lipid Metabolism (Figure 4B). Despite differences in the specific miRNA identities detected in each compartment, overlap at the functional category level was observed.

### 2.6. Comparative Analysis of Temporal miRNA Profiles Between Endometrial Tissue and Plasma

**Figure 5:**
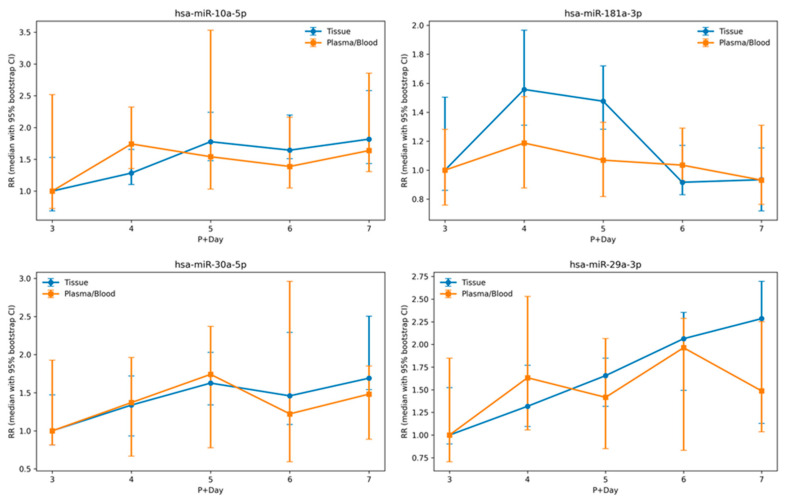
Comparative temporal expression patterns of selected miRNAs in endometrial tissue and plasma. Among temporally regulated miRNAs identified in endometrial tissue (DESeq2, adjusted *p* < 0.05), ten exhibited the same directional temporal trends in plasma across progesterone exposure days (P+3 to P+7). Four miRNAs—hsa-miR-10a-5p, hsa-miR-181a-3p, hsa-miR-30a-5p, and hsa-miR-29a-3p—exhibited correlated temporal trajectories between tissue and plasma based on Spearman correlation analysis (ρ > 0.4). Blue lines represent relative expression levels in endometrial tissue, and orange lines represent relative expression levels in plasma. Expression values are shown as relative fold changes with 95% bootstrap confidence intervals.

To evaluate temporal relationships between endometrial tissue and plasma miRNA expression, we performed a cross-compartment comparative analysis of temporally dynamic miRNAs identified in endometrial tissue and assessed their expression trajectories in plasma. Among tissue-defined temporally regulated miRNAs, ten exhibited the same directional temporal trends in plasma across progesterone exposure days (P+3 to P+7) (Supplementary Figure S4). Of these, four miRNAs demonstrated correlated temporal trajectories between tissue and plasma based on Spearman correlation analysis (ρ > 0.4) (Figure 5). The remaining six miRNAs with concordant directional trends but lower correlation coefficients are presented in Supplementary Table S5.

The four miRNAs exhibiting both directional agreement and higher correlation strength were miR-30a-5p, miR-181a-3p, miR-29a-3p, and miR-10a-5p. Functional annotation indicated that these miRNAs were primarily associated with Apoptosis (4/4) and Aging (3/4) categories, and additional shared functional categories are summarized in Table 2.

## 3. Discussion

The present time-resolved integrative analysis of paired endometrial tissue and plasma samples across progesterone exposure days (P+3 to P+7) suggests that temporally associated miRNA regulation during the peri-implantation window exhibits overlap at the level of enriched biological pathways despite divergence at the level of individual miRNA species. By implementing a hormonally controlled and temporally aligned sampling framework, this study enables cross-compartment evaluation of enrichment patterns within a defined physiological window. Importantly, the observed findings should be interpreted as enrichment-based associations rather than direct mechanistic concordance.

Although the sets of temporally dynamic miRNAs identified in endometrial tissue and plasma differed in composition and abundance, recurrent enrichment of apoptosis-, cell cycle-, aging-, inflammatory-, and metabolic-related functional categories was observed in both compartments under identical statistical criteria (FDR < 0.05). These shared enrichment patterns are consistent with the possibility that peri-implantation remodeling involves coordinated biological processes that may be detectable locally in the endometrium and systemically in circulation. However, such observations reflect functional category overlap rather than evidence of direct regulatory mirroring between compartments.

Endometrial tissue exhibited a higher proportion of miRNA reads and greater species diversity compared with plasma, consistent with sampling of localized epithelial, stromal, and immune cell populations. Temporally dynamic endometrial miRNAs were enriched in categories related to apoptosis, cell cycle regulation, epithelial–mesenchymal transition (EMT), inflammation, and lipid metabolism. These enrichment themes align with previously described biological processes associated with peri-implantation remodeling, including epithelial restructuring, decidual transformation, immune adaptation, and metabolic reprogramming within a progesterone-regulated window [14,15,16]. Nevertheless, the present analysis identifies enrichment-level associations and does not establish causal regulatory interactions.

In plasma, fewer temporally dynamic miRNAs were identified, and overlap at the individual miRNA level between compartments was limited. Despite this molecular divergence, enrichment analysis revealed overlap in apoptosis-, aging-, cell cycle-, inflammatory-, and metabolic-related categories. Apoptosis and regulated cell cycle activity have been described as features of decidual remodeling, and physiological senescence has been reported during luteal-phase transformation [17,18,19]. Within this context, the observed cross-compartment enrichment overlap may be compatible with temporally aligned biological remodeling rather than direct tissue-to-plasma molecular transmission.

Four miRNAs—miR-30a-5p, miR-181a-3p, miR-29a-3p, and miR-10a-5p—exhibited concordant directional temporal trends between tissue and plasma, with moderate correlation coefficients. These miRNAs were associated with apoptosis- and aging-related functional categories in enrichment analysis. While such concordance may suggest potential cross-compartment regulatory associations, the limited overlap in specific miRNA identities indicates that circulating profiles should not be interpreted as direct replicas of endometrial expression. A pathway-centric interpretation may therefore provide a complementary framework for examining cross-compartment regulatory associations in dynamic physiological systems.

The recurrent enrichment of apoptosis-, cell cycle-, inflammatory-, and metabolic-related categories across compartments may be compatible with coordinated endocrine–immune–metabolic remodeling during progesterone-driven endometrial transformation. However, these findings represent statistical enrichment overlap and should not be interpreted as proof of integrated systemic regulatory mechanisms. Circulating miRNAs may partially capture systemic signatures associated with reproductive remodeling, but causal pathways remain to be established.

Several limitations should be acknowledged. The study design was cross-sectional rather than longitudinal within individuals; therefore, temporal patterns were reconstructed across stratified groups rather than tracked repeatedly in the same subjects. Although hormonally controlled cycles enabled precise temporal alignment by progesterone exposure day, individual-level temporal trajectories were not obtained. In addition, the study was conducted exclusively in hormonally controlled cycles, and direct comparison with natural-cycle cohorts was not performed. Therefore, extrapolation of these findings to natural menstrual cycles should be made with caution. Furthermore, enrichment overlap was defined based on statistically significant functional categories identified independently in each compartment; formal permutation-based quantification of overlap probability was not performed. The sample size was sufficient for enrichment-level analysis, but detection of subtle molecular concordance may require larger cohorts. Implantation outcomes were not incorporated, and thus predictive or clinical inference cannot be drawn. Functional validation experiments were also beyond the scope of this study.

Despite these limitations, the present work establishes a temporally aligned analytical framework for evaluating cross-compartment miRNA-associated enrichment patterns during the human peri-implantation window. The findings should be interpreted within a hypothesis-generating context and provide a pathway-centric perspective that may guide future longitudinal, mechanistic, and quantitatively modeled investigations of implantation-related regulatory dynamics.

## 4. Materials and Methods

### 4.1. Ethical Approval

The study protocol was approved by the Institutional Review Board of Chung Shan Medical University Hospital (IRB No. CS2-22033). All participants were enrolled after providing written informed consent.

### 4.2. Study Population

This study was conducted at Lee Women’s Hospital (Taichung, Taiwan) between July 2022 and June 2024. Inclusion criteria were: (1) age between 25 and 38 years; (2) regular menstrual cycles of 28–32 days; (3) body mass index (BMI) between 18 and 30 kg/m^2^; and (4) serum progesterone (P4) level < 1 ng/mL prior to hormone administration. Exclusion criteria included suspected uterine abnormalities, breastfeeding, a history of pelvic inflammatory disease, reproductive tract disorders, sexually transmitted infections, systemic diseases, endocrine disorders, or other major medical conditions, as well as use of hormonal contraceptives or intrauterine devices within the preceding three months.

A total of 62 participants meeting the eligibility criteria were recruited and assigned to study groups according to progesterone exposure days (P+3 to P+7). All participants initiated oral estradiol valerate treatment (Estrade^®^; Synmosa, Hsinchu, Taiwan) at a dose of 6 mg/day beginning on cycle day 2. Hormonal evaluation was performed on cycle days 10–12. After confirmation that serum P4 remained <1 ng/mL, daily subcutaneous progesterone (Prolutex^®^, IBSA, Paradiso, Switzerland) was initiated.

The duration of progesterone administration ranged from 3 to 7 consecutive days, corresponding to progesterone exposure days P+3 through P+7 prior to sample collection. Endometrial tissue and peripheral blood samples were collected 24 h after the final progesterone administration on the designated progesterone exposure day (P+3 to P+7). The clinical characteristics, including serum progesterone and estradiol levels as well as endometrial thickness at each progesterone exposure day (P+3 to P+7), are summarized in Table 1.

### 4.3. Endometrial Tissue and Plasma Sample Collection and Preparation

Endometrial tissue samples were obtained using a Pipelle catheter (UNIMAX, New Taipei City, Taiwan; Cat. No. FEM103600) and immediately preserved in RNAlater solution (Thermo Fisher Scientific, Waltham, MA, USA; Cat. No. AM7022) according to the manufacturer’s instructions.

Peripheral blood samples (5–10 mL per participant) were collected into EDTA anticoagulant tubes (BD, Mississauga, ON, Canada; Cat. No. 367525). All samples across progesterone exposure groups (P+3 to P+7) were collected, processed, and stored using an identical standardized protocol to minimize pre-analytical variability.

Following collection, tubes were gently inverted at least five times and processed within 60 min. Samples were centrifuged at 1200× *g* for 10 min at room temperature to separate plasma from cellular components. The plasma supernatant was carefully transferred to new RNase-free tubes and centrifuged again at 12,000× *g* for 10 min to remove residual cellular debris. The clarified plasma was aliquoted into RNase-free tubes and stored at −80 °C until further analysis.

### 4.4. Small RNA Extraction

Total RNA, including small RNAs, was extracted from approximately 5 mg of endometrial tissue using the miRNeasy Micro Kit (QIAGEN, Hilden, Germany; Cat. No. 217804) and from 600 μL of clarified plasma using the miRNeasy Serum/Plasma Advanced Kit (QIAGEN; Cat. No. 217204), according to the manufacturer’s instructions. RNA was eluted in nuclease-free water.

Small RNA concentration was quantified using the Qubit microRNA Assay Kit (Thermo Fisher Scientific; Cat. No. Q32880), which selectively measures the microRNA-enriched small RNA fraction. For each sample, 10 ng of miRNA, as determined by the Qubit microRNA assay, was used as input for library preparation. All samples yielded sufficient small RNA quantities for downstream library construction.

### 4.5. miRNA Library Construction and Sequencing

miRNA sequencing libraries were constructed using the QIAseq miRNA Library Kit (QIAGEN; Cat. No. 331502) according to the manufacturer’s protocol. Briefly, library preparation included: (1) 3′ adapter ligation using a pre-adenylated adapter; (2) 5′ adapter ligation; (3) reverse transcription using primers containing unique molecular identifiers (UMIs) to enable molecule-level quantification; (4) cDNA cleanup; (5) PCR amplification using primers with sample-specific barcodes; and (6) final library purification. Library quality and fragment size distribution were assessed using the Agilent 5200 Fragment Analyzer System (Agilent Technologies, Santa Clara, CA, USA). The expected library fragment size ranged from approximately 190 to 220 bp. Libraries were quantified using the Qubit microRNA Assay Kit (Thermo Fisher Scientific), and only libraries with concentrations greater than 1 ng/μL were subjected to sequencing. Sequencing was performed using single-end 75 bp reads on an Illumina NextSeq 550 platform (Illumina, San Diego, CA, USA) in accordance with the manufacturer’s protocols.

### 4.6. NGS Data Analysis Pipeline

An NGS data analysis pipeline was established for miRNA expression profiling. Raw sequencing data (FASTQ format) were first subjected to quality control, including adapter trimming and removal of low-quality reads, using FastQC (version 0.11.9) [20] and Trimmomatic (version 0.39) [21]. Reads with low-quality bases (Phred quality score < 20) at either end were trimmed, and reads shorter than 17 bp or longer than 55 bp after trimming were discarded.

The remaining high-quality reads were aligned to the human reference genome (GRCh38/hg38) using the Bowtie aligner (version 1.3.1) [22] and to annotated small RNA sequences obtained from miRbase (version 22.1) [23]. Aligned reads were subsequently processed and quantified using SAMtools (version 1.14) [24], and miRNA annotation was performed based on miRBase reference annotations (release containing 2656 human miRNAs). For each sample, the read counts mapped to individual miRNAs were extracted and used as expression values for downstream analyses, including differential expression and pathway-level analyses.

### 4.7. Identification of Dynamic and Differentially Expressed miRNAs

Samples were collected across five consecutive progesterone exposure days (P+3 to P+7). Time-dependent miRNA expression was analyzed using DESeq2 (v1.38.0), which models raw count data using a negative binomial generalized linear model. Size factor normalization (median-of-ratios method) and dispersion estimation were performed using DESeq2 default parameters. No additional batch correction was applied. As specified by the DESeq2 workflow, raw count data were used as input for all statistical analyses.

To identify miRNAs exhibiting significant global temporal effects across progesterone exposure days, a likelihood ratio test (LRT) was performed by comparing a full model including time (~day) with a reduced intercept-only model (~1). miRNAs with Benjamini–Hochberg adjusted *p*-values (false discovery rate, FDR) < 0.05 were defined as temporally dynamic miRNAs.

For descriptive purposes, Wald tests were conducted for pairwise comparisons between adjacent time points (P+4 vs. P+3, P+5 vs. P+4, P+6 vs. P+5, and P+7 vs. P+6) to estimate effect sizes and determine the directionality of temporal changes. Log2 fold changes were reported for these comparisons. No independent filtering was applied prior to statistical testing [25].

### 4.8. Functional Enrichment Analysis

Temporally dynamic miRNAs identified as described in Section 4.7 were subjected to functional enrichment analysis using TAM 2.0 (Tool for MicroRNA Set Analysis) [26]. Enrichment analysis was performed to identify overrepresented biological functions and miRNA-associated functional categories within the temporally regulated miRNA sets. Statistical significance was defined as FDR < 0.05.

### 4.9. Correlation Analysis Between Clinical Characteristics and Temporally Dynamic miRNAs

Correlation analyses were performed using two complementary approaches.

Pearson correlation coefficients were calculated to evaluate associations between clinical characteristics and temporally dynamic endometrial miRNAs. The corresponding *t* statistic was calculated as:

$$t=\frac{r}{\sqrt{\frac{1-r^{2}}{n-2}}}$$

where *r* represents the correlation coefficient and *n* denotes the sample size. Two-tailed *p*-values were derived accordingly [27].

For cross-compartment analyses, Spearman rank correlation was used to assess similarity in temporal expression trajectories between endometrial tissue and plasma miRNAs across progesterone exposure days (P+3 to P+7). miRNAs with Spearman correlation coefficients (ρ) > 0.4 were considered to exhibit concordant temporal patterns between compartments.

## 5. Conclusions

This temporally aligned integrative analysis suggests that dynamic miRNA-associated enrichment patterns during the human peri-implantation window may exhibit overlap at the pathway level despite divergence in individual miRNA composition. Recurrent enrichment of apoptosis-, cell cycle-, aging-, inflammatory-, and metabolic-related functional categories across compartments reflects statistical enrichment-based associations rather than direct mechanistic concordance. By adopting a pathway-centric and time-resolved framework, this study provides a hypothesis-generating perspective for future systems-level and longitudinal investigations of implantation-related regulatory networks.

## 6. Patents

The patent has been filed as a provisional application.

## Figures and Tables

**Table 1:** Clinical and Hormonal Characteristics of Participants in the Hormone Replacement Therapy (HRT) Cycle Stratified by Days after Progesterone Administration (P+3 to P+7).

Variable		Time Point	P+3 (*n* = 12)	P+4 (*n* = 12)	P+5 (*n* = 13)	P+6 (*n* = 13)	P+7 (*n* = 12)
Age (years)	–	31.3 ± 4.6	29.2 ± 2.8	30.3 ± 4.1	31.6 ± 4.7	30.6 ± 3.7	
BMI (kg/m^2^)	–	21.9 ± 2.1	22.1 ± 1.9	22.6 ± 3.7	21.4 ± 2.4	22.3 ± 2.4	
Previous Pregnancy, *n*	–	7	3	5	9	6	
No Previous Pregnancy, *n*	–	5	9	8	4	6	
FSH (mIU/mL)	Day 2	6.28 ± 1.90	6.16 ± 1.45	8.09 ± 2.43	7.25 ± 2.04	6.50 ± 1.78	
	Day 10–12	6.19 ± 2.16	6.05 ± 2.14	6.33 ± 1.63	6.33 ± 2.08	6.15 ± 1.24	
	P+Day	3.30 ± 1.37	3.88 ± 1.33	4.14 ± 1.90	3.26 ± 0.89	4.02 ± 1.61	
LH (mIU/mL)	Day 2	4.19 ± 1.43	4.09 ± 1.40	4.05 ± 1.79	4.81 ± 3.86	4.83 ± 3.23	
	Day 10–12	15.78 ± 8.78	14.09 ± 9.49	11.98 ± 7.12	15.53 ± 7.42	16.74 ± 7.58	
	P+Day	4.66 ± 2.12	5.36 ± 2.91	5.08 ± 2.24	7.00 ± 3.55	6.99 ± 4.24	
Estrogen (pg/mL)	Day 2	42.83 ± 29.66	49.67 ± 16.91	52.31 ± 19.86	48.00 ± 26.36	50.17 ± 23.51	
	Day 10–12	503.42 ± 187.38	462.17 ± 137.92	505.54 ± 259.17	616.08 ± 317.34	535.17 ± 261.35	
	P+Day	376.58 ± 192.60	238.17 ± 94.36	353.23 ± 179.89	363.08 ± 190.00	336.08 ± 213.03	
Progesterone (ng/mL)	Day 2	0.33 ± 0.25	0.39 ± 0.31	0.67 ± 0.57	0.32 ± 0.17	0.48 ± 0.20	
	Day 10–12	0.30 ± 0.21	0.28 ± 0.14	0.34 ± 0.20	0.38 ± 0.24	0.37 ± 0.24	
	P+Day	3.82 ± 1.50	3.90 ± 1.46	3.05 ± 1.33	3.55 ± 1.70	3.06 ± 1.34	
Endometrial Thickness (mm)	Day 2	5.55 ± 1.22	7.33 ± 2.89	6.55 ± 2.08	6.65 ± 2.21	6.46 ± 1.78	
	Day 10–12	9.43 ± 1.69	9.78 ± 1.98	10.00 ± 2.67	10.27 ± 1.60	12.15 ± 2.16	
	P+Day	10.47 ± 2.53	10.18 ± 2.14	11.24 ± 2.92	11.38 ± 2.32	12.13 ± 3.21	

P+Day indicates the number of days after initiation of progesterone administration in the hormone replacement therapy (HRT) cycle. Data are presented as mean ± standard deviation (SD) unless otherwise indicated. Hormonal measurements were obtained on cycle Day 2, Day 10–12, and the corresponding progesterone exposure day (P+Day). Sample numbers indicate the number of participants included in each group. FSH, follicle-stimulating hormone; LH, luteinizing hormone; BMI, body mass index.

**Table 2:** Shared Enriched Functional Categories between Temporally Dynamic miRNAs in Endometrial Tissue and Plasma.

Function	Tissue	Plasma	miR-30a-5p	miR-181a-3p	miR-29a-3p	miR-10a-5p
Cell Death	miR-29c, miR-30d, miR-181a, miR-30b, miR-29b, miR-182, miR-183, miR-205, miR-29a	miR-24, miR-7, let-7f, miR-885, miR-10b		✓	✓	
Apoptosis	miR-181a, miR-29b, miR-449a, miR-30a, miR-10a, miR-182, miR-29c, miR-30b, miR-15b, miR-29a	miR-24, miR-7, miR-323a, miR-122	✓	✓	✓	✓
Cell Cycle	miR-15b, miR-29b, miR-191, miR-182, miR-449a, miR-205	miR-24, miR-31, miR-122, miR-331				
Aging	miR-30d, miR-181a, miR-30a, miR-30b, miR-10a	miR-7, miR-31, miR-122, miR-511	✓	✓		✓
Inflammation	miR-181a, miR-182, miR-30b, miR-183, miR-205, miR-29a	miR-24, miR-7, miR-122, let-7f, miR-31		✓	✓	
Tumor Suppressor miRNAs	miR-29c, miR-181a, miR-30b, miR-29a	miR-7, let-7f, miR-122		✓	✓	
Lipid Metabolism	miR-29c, miR-224, miR-29a	miR-10b, miR-122, miR-375			✓	

Temporally dynamic miRNAs were identified using DESeq2 (adjusted *p* < 0.05) and subjected to TAM 2.0 functional enrichment analysis (FDR < 0.05). The “Tissue” and “Plasma” columns list miRNAs contributing to enrichment within each compartment. The columns miR-30a-5p, miR-181a-3p, miR-29a-3p, and miR-10a-5p indicate whether miRNAs exhibiting concordant temporal expression patterns in both tissue and plasma are involved in the corresponding functional category (✓ = present; blank = not detected).

## Data Availability

The datasets generated and analyzed during this study are available on request from the corresponding author and in the Gene Expression Omnibus (GEO) database with accession number GSE297808.

## Supplementary Materials

The following supporting information can be downloaded at: https://www.mdpi.com/article/10.3390/ijms27052414/s1.

## Abbreviations

The following abbreviations are used in this manuscript:

BMI	Body mass index
bp	Base Pair
EMT	Epithelial-to-Mesenchymal Transition
FDR	False Discovery Rate
FSH	Follicle stimulating hormone
HRT	Hormone replacement therapy
LH	Luteinizing hormone
miRNAs	microRNAs
NGS	Next-Generation Sequencing
P4	Progesterone
P+Day	Days after Initiation of Progesterone Administration
TAM	Tool for MicroRNA Set Analysis
UMI	Unique molecular index
WOI	Window of implantation
