# Supplementary material for: Pathway-Level Convergence Between Dynamic Plasma miRNAs and Endometrial Biological Processes During the Human Peri-Implantation Window
Source: Int J Mol Sci. 2026 Mar 5;27(5):2414. doi: 10.3390/ijms27052414 (PMC12986078; doi:10.3390/ijms27052414)
Supplement: Supplementary file 1 [file ijms-27-02414-s001.zip › supplementary file-0225.pdf]

Supplementary Materials

Supplementary Figure S1. Overview of the Study Design.

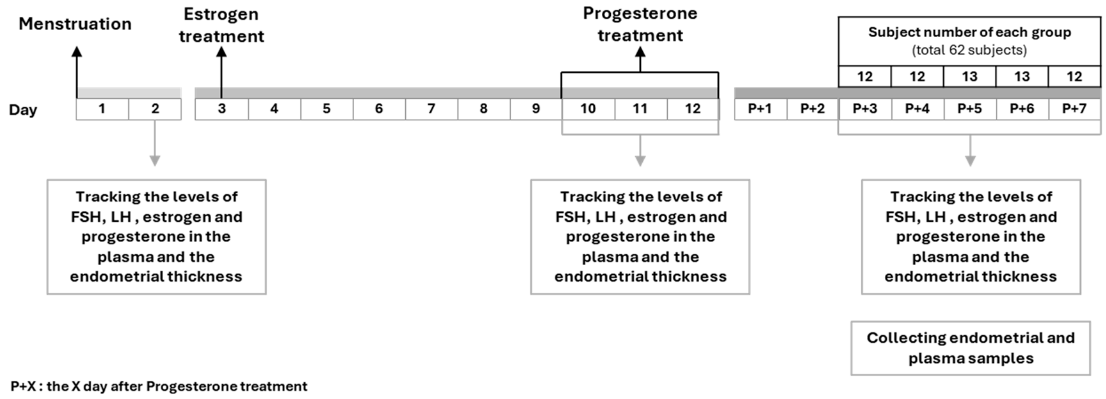

Participants underwent a hormone replacement therapy (HRT) cycle. Oral estradiol valerate (6 mg/day) was administered from day 2 of the menstrual cycle, and serum hormone levels and endometrial thickness were measured on day 10–12. Upon confirming a serum progesterone (P4) level < 1 ng/mL, daily subcutaneous progesterone injections (Prolutex, IBSA, Switzerland) were initiated. All participants began progesterone on the same day; however, the duration of progesterone exposure varied according to the assigned sampling day (P+3 to P+7). Endometrial tissue and peripheral blood samples were collected immediately after the final progesterone dose on the designated P+ day.

Supplementary Figure S2. Changes in Clinical Characteristics during the Peri-Implantation Period.

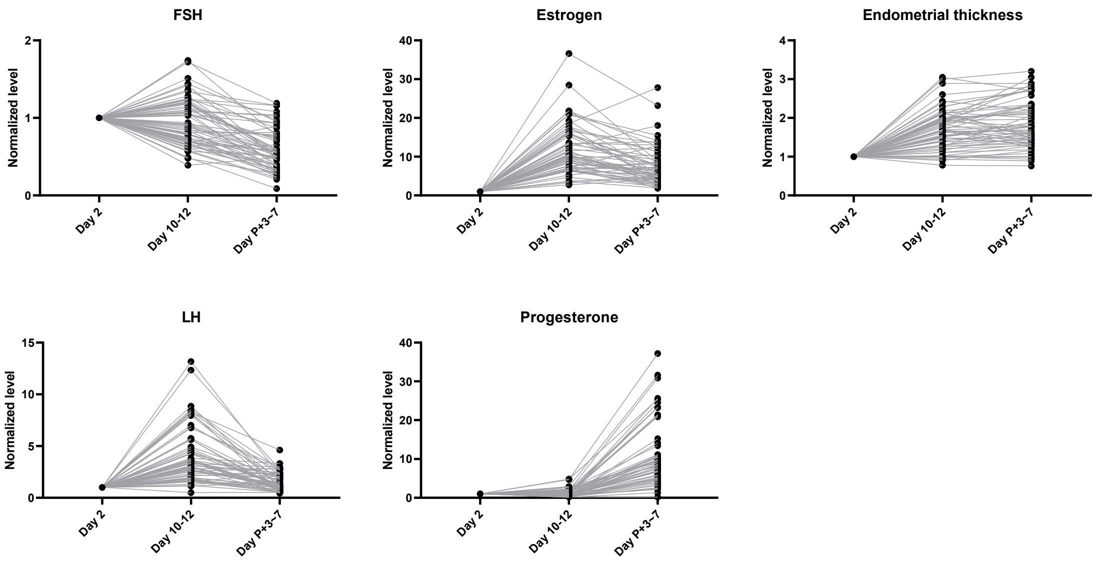

The x-axis represents sampling time points, and the y-axis reflects relative levels compared with day 2.

Supplementary Figure S3. Correlation Analysis between Clinical Characteristics and Temporally Dynamic Endometrial miRNAs.

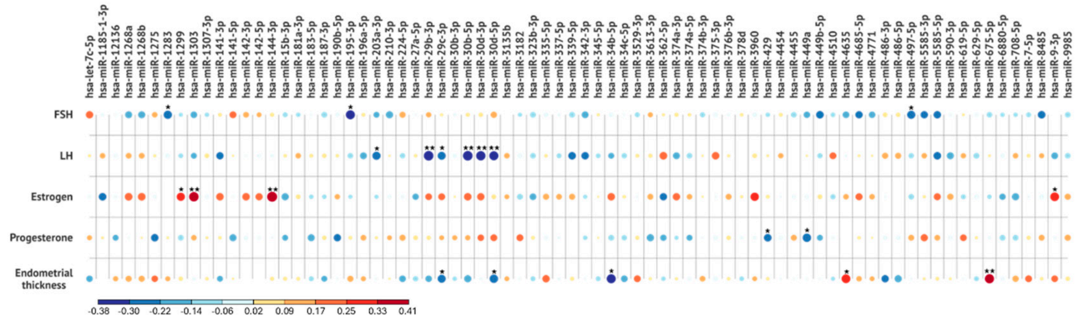

The x-axis represents temporally dynamic endometrial miRNAs, and the y-axis shows clinical characteristics. Yellow and red dots indicate positive correlations, whereas light and dark blue dots represent negative correlations. Dot size corresponds to statistical significance (* for $0.01 < p < 0.05$; ** for $p < 0.01$).

Supplementary Figure S4. Correlation Analysis between Endometrial Tissue and Plasma miRNAs Exhibiting Concordant Temporal Trends.

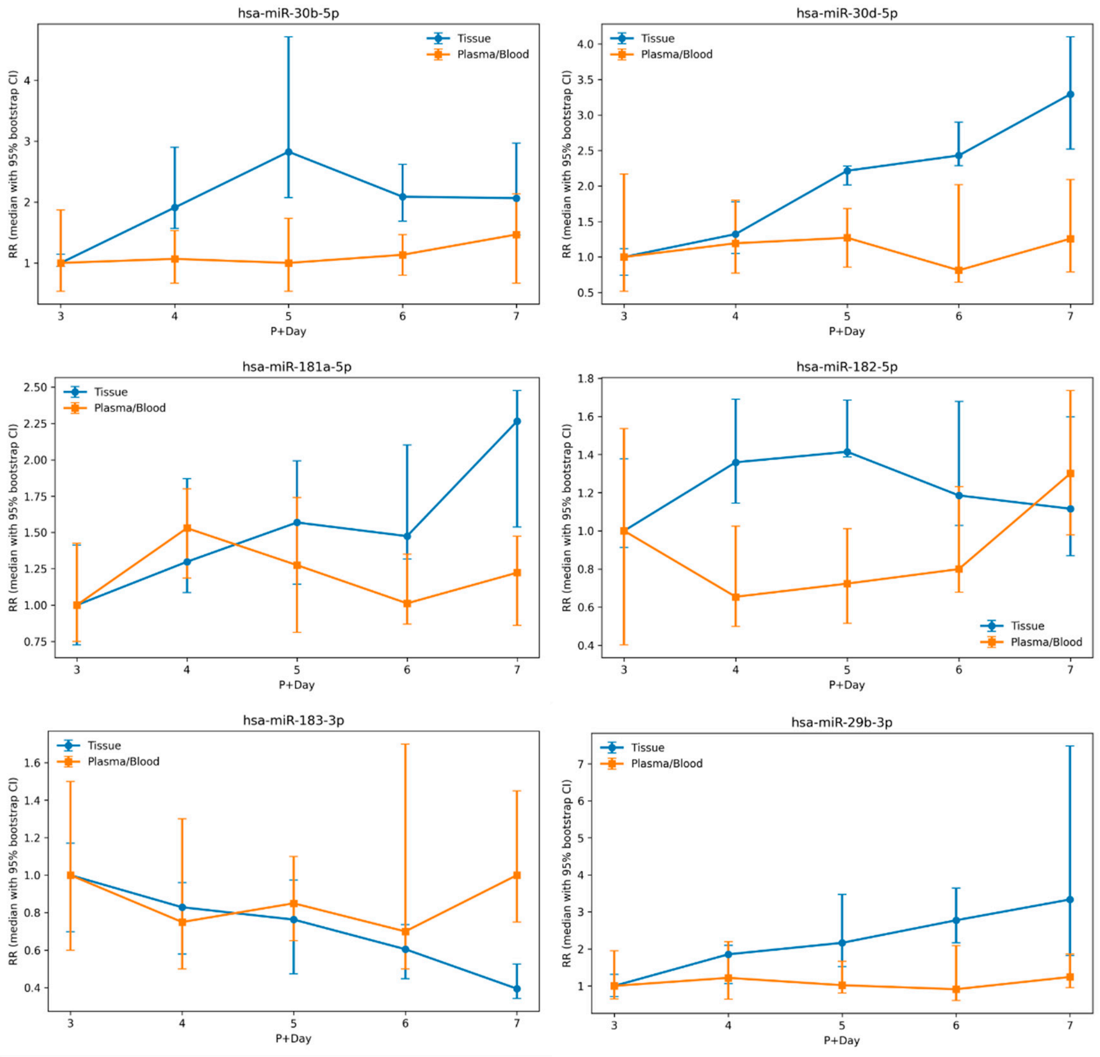

Scatter plots illustrate the correlation of expression levels between matched endometrial tissue and plasma samples for miRNAs exhibiting consistent temporal patterns across compartments.
